# Effect of delayed entry of blood culture bottles in BACTEC automated blood culture system in the context of laboratory consolidation

**DOI:** 10.1038/s41598-022-05246-3

**Published:** 2022-01-25

**Authors:** Vincent Deslandes, Darya Rafipour, Ivan Gorn, Elham Sabri, Nadia Sant, Marc Desjardins

**Affiliations:** 1 University of Ottawa, Ottawa, ON Canada; 2 The Ottawa Hospital Research Institute (OHRI), Ottawa, ON Canada; 3 Eastern Ontario Regional Laboratory Association (EORLA), Ottawa, ON Canada

**Keywords:** Microbiology, Clinical microbiology, Infectious-disease diagnostics

## Abstract

Delayed entry of blood culture bottles is frequent in consolidated laboratories. A retrospective study evaluated time from insertion to detection and total detection time as a function of preincubation time, and we prospectively looked for false negative results. 69,604 blood culture bottles were reviewed for preincubation time, incubation time and total detection time. Positive cultures for specific bacterial subtypes were reviewed to assess the effect of preincubation time on likelihood of detection. 492 negative blood cultures were prospectively tested by 16S RNA PCR and *Staphylococcus*-specific PCR for the presence of bacterial DNA. Mean preincubation time for samples collected within the city-limits was 3.94 h versus 9.49–18.89 h for other client sites. Higher preincubation times were partially mitigated by a lower incubation time, with an overall increase in total detection time. A lower odds ratio of recovery of *Staphylococcus* spp was identified, but not confirmed by terminal subcultures and molecular assays. Prolonged preincubation of blood cultures affects total detection time despite a reduction in incubation time. Successful centralization of microbiological services may depend upon optimization of courier routes for inoculated blood culture bottles. Our data supports consideration for an increase in suggested maximum preincubation times.

## Introduction

Laboratory consolidation in the healthcare system has been a reality since the 1970s, and the phenomenon has increased over time with core laboratories, often located in tertiary centers, offering microbiology diagnostic services to surrounding smaller community hospitals. These efforts have been driven by both expected cost efficiency and improvement in patient care^1,2^. As part of the consolidation process, decisions need to be made regarding what test, if any, should be kept at the peripheral sites, including blood cultures. Previous studies have shown that an increase in transport time, while accompanied by a reduction in incubation time, is linked with an overall increase in total detection time of the etiological agents responsible for episodes of bacteremia^3–6^. It has been previously established that timely reporting of positive blood cultures can impact the overall use of antibiotics^7^, the adequacy of the antimicrobial coverage^8^, and overall patient survival during septic episodes^9^. As a result, some expert opinion supports maintaining the provision of blood cultures at peripheral sites^1^. Currently, it is recommended that blood recovered from patients for the purpose of culture be kept at room temperature while awaiting incubation, which should occur within 4 h of collection^10,11^. The Clinical and Laboratory Standards Institute (CLSI) provides a more stringent guideline, recommending that inoculated blood culture bottles be sent to the laboratory within 2 h, and that they should not be held at room temperature for “anything longer than a few hours”^12^. This guideline does not however list delayed entry into the continuous-monitoring blood culture system as a criterion for sample rejection, and further clarifies that delays do not impact bacterial recovery but may prolong overall time to detection. Finally, preincubation of delayed blood culture bottles at 35-37ºC prior to entry in continuous-monitoring blood culture system is not recommended, and neither is the storage of inoculated bottles at 4ºC^13^.

These suggestions all aim to maximize bacterial recovery and avoid the reporting of false negative results. However, a diminution in bacterial recovery has only been documented following > 24 h delayed entry for bottles held at room temperature, mainly for isolates of *Streptococcus pneumoniae* after delayed entry > 12 h with the BACTEC system^5^.

The Eastern Ontario Regional Laboratory Association (EORLA) has been consolidating microbiology laboratory services in eastern Ontario since 2011, and currently provides testing for 19 hospitals within an 18,000 km sq. area, with a population of 1.3 million residents. As part of the service offering, blood cultures were also centralized to the Regional Microbiology Reference Laboratory (RMRL) and are incubated in the BACTEC FX continuous-monitoring system. As part of a quality improvement initiative and in order to assess the effect of transport time on incubation time and total time to positivity, we conducted a retrospective one-year study of the 69,604 blood culture bottles handled by the core-laboratory. Based on our findings, we prospectively tested inoculated blood culture bottles with delayed entry > 12 h and no growth at 5 days in BACTEC FX for the presence of undetected bacterial growth.

## Results

### Preincubation time, INC and TDT for each site

**Figure 1:**
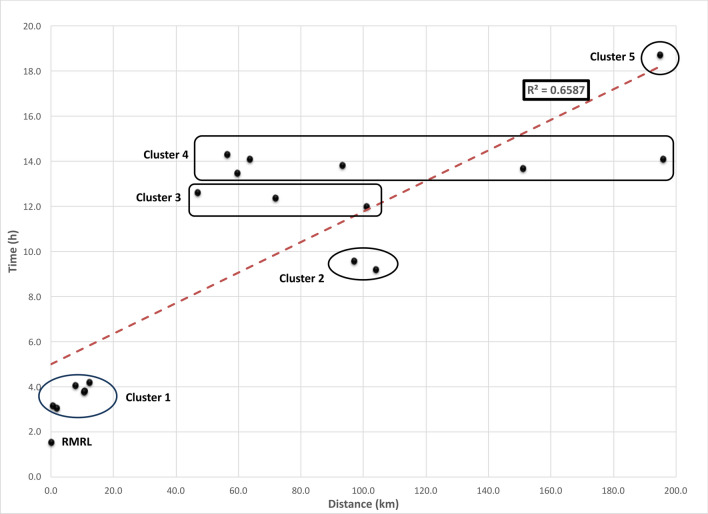
Median preincubation time as a function of distance travelled. Sites with similar median preincubation times were clustered as shown on the graphic.

**Figure 2:**
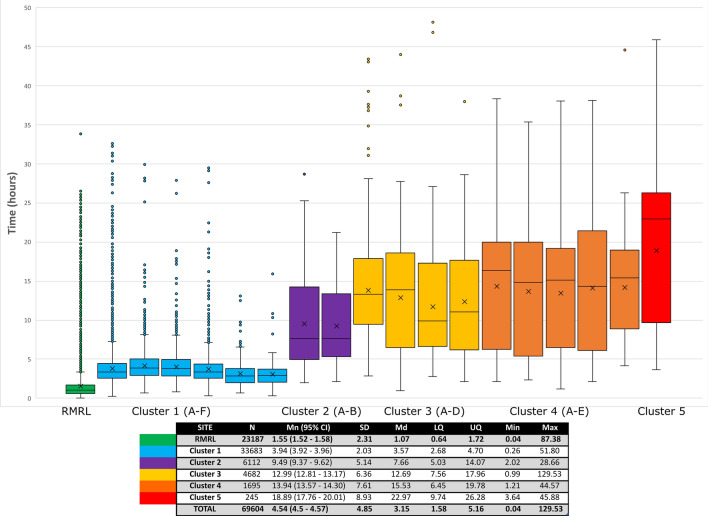
Distribution of preincubation times (h) per sites, showing interquartile ranges. Data points between 0 and 50 h are represented on the figure. Data series are presented in alphanumerical order of clusters and sites. Outlier points as per the 1.5 × IQR rule are included on the graphic. X: mean. Inset: mean preincubation times (h) for all sites clusters with standard deviation, median and lower/upper quartile). N: number of samples, Mn: mean, CI: confidence interval, SD: standard deviation, Md: median, LQ: lower quartile, UQ: upper quartile, Min: minimum, Max: maximum.

A total of 69,604 positive (10,527) and negative (59,077) blood culture bottles were assessed. Mean and median preincubation times are presented in Table 1. An average preincubation time of 4.54 h per blood culture bottle was calculated (Fig. 2). Blood cultures collected at the testing site were inserted in the automated blood culture system on average 1.5 h after collection, compared to 18.7 h for cultures collected at one of the most distant sites. For this site, which has one daily courier run, nearly 40% of blood culture bottles had a preincubation time of more than 24 h. All sites within the Ottawa city limits had 8 or more daily courier pick up time, whereas community sites had between 1 and 4 daily couriers (Table 1). Of the culture bottles referred in, 0.19% were delayed > 24 h. Sites in cluster 2 (Fig. 2), located approximately 100 km away from the testing site, had 3 and 4 daily pick ups with mean preincubation times of 9.2 and 9.6 h, whereas some sites from cluster 4 which were either equidistant or closer to the testing site but had fewer daily pick ups recorded mean preincubation times between 13.8 and 14.3 h. Median preincubation time increased as a function of the distance traveled, which explained approximately 60% of the variation noted between sites (R^2^ = 0.593) (Fig. 1). Distribution of preincubation times for each site showed greater variability for smaller community sites (clusters 3 to 5) when compared to inner city sites and larger community centers (clusters 1 and 2) (Fig. 2). The mean preincubation time was statistically significantly different across all cluster sites (*P* < 0.0001).

**Table 1:** Preincubation times for negative blood culture bottles for all study sites and incubation (INC) and total detection times (TDT) for positive blood culture bottles for all study sites.

SITE	Baseline characteristics			Negative blood culture bottles					Positive blood culture bottles				
	Dist. (km)	Hours	Runs	N	Mn	Md	TT90%	> 24 h	N	POS %	CONT %	Mn INC	Mn TDT
RMRL site	0	24 h	–	19,610	1.5	1.1	2.6	0.14	3577	12.92	2.5	21.31	22.99
Cluster 1, site A	10.9	24 h	12	11,736	3.8	3.4	5.9	0.19	2316	13.38	3.1	21.39	25.18
Cluster 1, site B	12.3	24 h	10	8533	4.2	3.9	6.5	0.11	1338	11.19	1.9	18.18	22.32
Cluster 1, site C	7.9	24 h	9	5373	4.0	3.8	6.2	0.04	1078	14.54	2.2	19.06	23.08
Cluster 1, site D	10.6	24 h	12	2129	3.8	3.4	5.7	0.14	348	10.4	3.7	**23.58**	27.24
Cluster 1, site E	0.5	6h30-22h00	13	540	3.1	2.8	5.6	0.00	61	7.14	3.0	22.78	25.85
Cluster 1, site F	1.8	24 h	8	214	3.1	2.9	4.6	0.00	17	**6.93**	0.4	17.61	**20.77**
Cluster 2, site A	97	24 h	4	4096	9.6	7.6	17.3	0.07	556	10.98	1.0	16.98	26.44
Cluster 2, site B	104	24 h	3	1277	9.2	7.6	16.7	0.00	183	10.55	2.0	19.44	29.27
Cluster 3, site A	151	24 h	3	1734	13.7	13.2	20.8	0.86	275	13.37	0.3	15.28	29.23
Cluster 3, site B	46.9	6h00-23h00	3	893	12.6	12.6	21.0	2.22	253	18.3	3.7	17.39	30.82
Cluster 3, site C	101	24 h	2	680	12.0	10.0	20.1	0.91	108	12.82	0.9	14.93	25.04
Cluster 3, site D	71.8	7h00-23h00	2	662	12.4	10.4	21.0	4.98	77	10.15	0.3	**10.14**	22.71
Cluster 4, site A	63.7	7h00-23h00	2	351	14.1	16.4	22.5	4.84	101	**20.58**	1.8	15.32	30.42
Cluster 4, site B	93.2	6h30-20h00	2	311	13.8	15.2	23.7	8.36	72	18.54	0.3	15.45	28.55
Cluster 4, site C	59.6	7h00-23h00	1	279	13.5	15.1	22.4	6.76	49	14.24	0.6	13.68	26.53
Cluster 4, site D	56.4	6h30-22h30	2	265	14.3	15.5	24.8	13.21	51	13.29	2.8	17.08	30.02
Cluster 4, site E	196	7h30-23h00	2	194	14.1	15.2	21.2	1.52	22	9.09	1.4	12.57	25.57
Cluster 5, site A	195	7h00-23h00	1	200	18.7	22.7	29.3	39.90	45	15.32	2.8	14.12	**32.66**

Time values are listed in hours and sites are presented in reverse order of testing volume. INC is defined as time from insertion into the automated blood system to growth detection and TDT is defined as time from collection to growth detection. The lowest and highest values for POS %, Mn INC and Mn TDT are presented in bold for emphasis. Dist: distance, N: number, Hours: hours of operation, Runs: daily number of couriers, Mn: mean, Md: median, TT90%: time to reception of 90% of samples, > 24 h: percentage of samples received after 24 h for each sites, POS %: monomicrobial blood culture positivity rate, CONT %: contamination rate.

**Figure 3:**
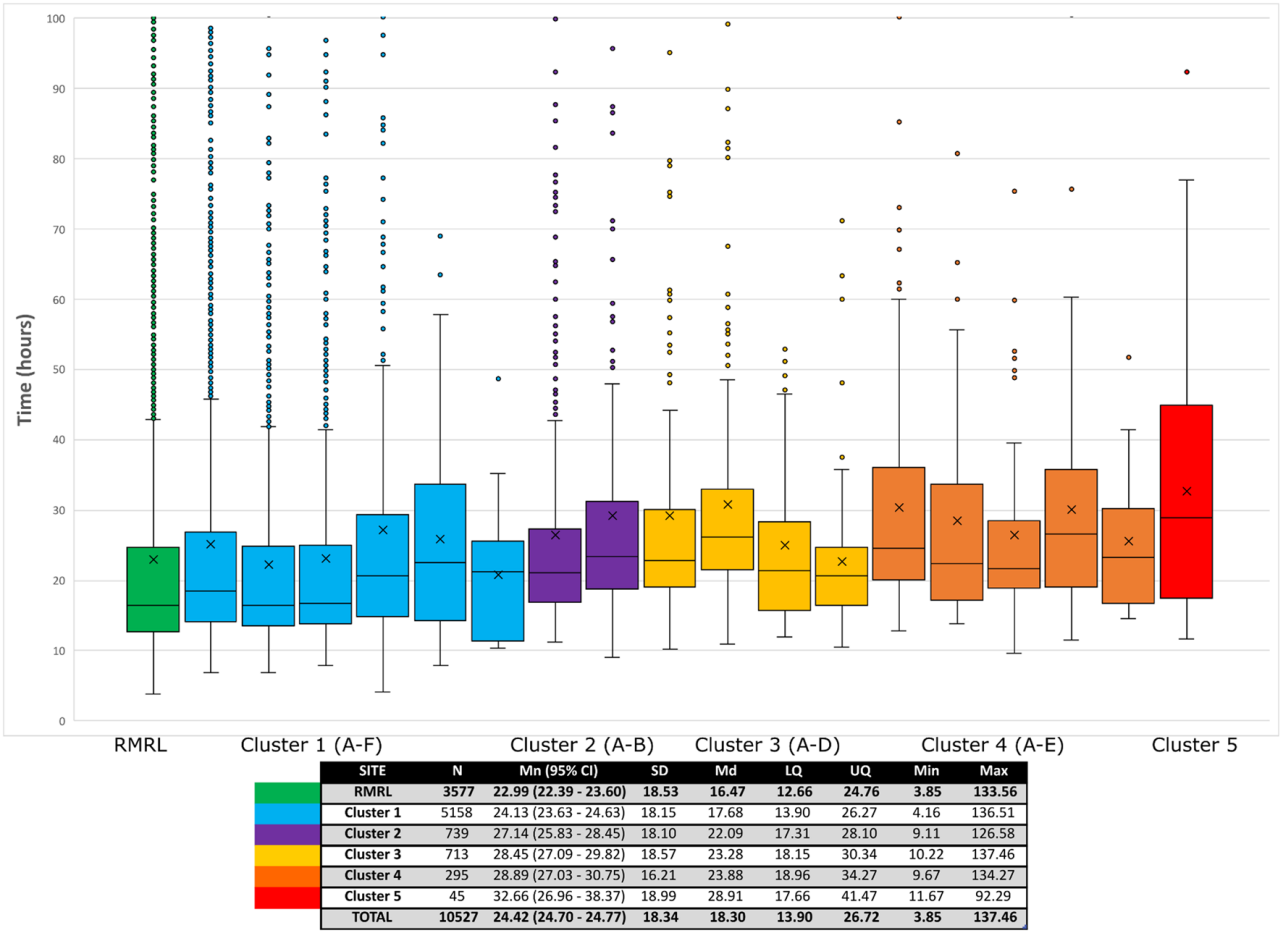
Distribution of total detection time (TDT) (h) per sites, showing interquartile ranges. Data points between 0 and 100 h are represented on the figure. Outlier points as per the 1.5 × IQR rule are included on the graphic. X: mean. Inset: mean TDT for all sites clusters along with standard deviation, median and lower/upper quartile. N: number of samples, Mn: mean, CI: confidence interval, SD: standard deviation, Md: median, LQ: lower quartile, UQ: upper quartile, Min: minimum, Max: maximum.

Distribution of TDT for all sites (Fig. 3) showed less spread than the distribution of preincubation times, and sites from clusters 3 to 5 tended to have lower INC (Table 1) than sites with shorter preincubation times. The shortest TDT was seen for one of the smaller inner-city sites with 20.7 h. TDT were statistically significantly different across all clusters (*P* < 0.0001), with a difference of 11.9 h between the site with the shortest TDT (20.7 h) and the site with the longest TDT at 32.67 h. Inner-city sites had on average an increase of 1.14 h in TDT as compared to the testing site (24.13 h). Stratification of cultures into four preincubation time clusters (Supplementary data, Table S2) showed non-overlapping confidence intervals for INC for time clusters < 5 h, 5–10 h and 10–20 h, but the confidence interval for time clusters > 20 h completely overlapped with the 10–20 h time cluster. An overall trend towards a decrease in INC with increasing preincubation times was noted but was not statistically significant (*P* = 0.08). While blood cultures with preincubation times < 5 h required on average 20.58 h of incubation, cultures with preincubation times > 10 h required approximately 16.14 h of incubation, a 4.44 h difference. There was no further reduction in mean INC with preincubation times > 20 h.

### Positivity rates as a function of preincubation time, and per bacterial species or groups

There was no statistically significant difference in positivity rates across all time clusters, and no clear trend in positivity rates was identified between time clusters (Table S2). Logistical regression analysis using the < 5 h time clusters as a reference showed no statistically significant decrease in the odds ratio of recovering growth in time clusters containing samples with preincubation times > 5 h. We also analyzed the full data set using logical regression and time as a continuous variable, yielding an OR of 0.998 (95% CI 0.994–1.003) for every additional hour of preincubation, not reaching statistical significance (*P* = 0.4456).

The same analysis was repeated after clustering positive cultures by bacterial species or groups, while keeping the same site clusters and preincubation time stratifications (Tables 2, 3). There were no beta-hemolytic *Streptococcus*, *S. aureus*, *viridans* group *Streptococcus*, *S. anginosus* group and *K. pneumoniae* recovered from the site with the highest mean preincubation times. There were significant differences noted in positivity rates between time clusters for *S. aureus*, coagulase-negative *Staphylococcus* (CoNS) and *E. coli*: trends towards lower positivity rates with increasing preincubation times for *S. aureus* and CoNS, and towards higher positivity rates with increasing preincubation times for *E. coli*. For *S. aureus*, logistic regression analysis showed significantly lower odds ratio for the 5–10 h (OR = 0.82) and > 20 h (OR = 0.50) time clusters when compared to the reference < 5 h time cluster, with an overall OR/h of 0.99 when using time as a continuous variable, which also reached statistical significance. For CoNS, logistic regression using time clusters yielded a significantly lower OR for the 10–20 h time cluster only (OR = 0.61), with an overall OR/h of 0.97 also reaching statistical significance. The opposite trend was noted for *E. coli*, with significantly higher OR at 10–20 h and > 20 h (OR = 1.65 and 2.15 respectively), and OR/h of 1.04. A significant increasing trend was also noted for other *Enterobacterales*, with a significantly higher OR at 10–20 h (OR = 1.34) and OR/h of 1.02. Of note, the small total number of anaerobic organisms (n = 101) and fastidious organisms such as *Haemophilus* spp (n = 42) or *Neisseria* spp (n = 5) recovered across all clusters and sites precluded analysis.

**Table 2:** Mean INC and TDT (h) for various bacterial species or groups per site clusters.

	Testing site	Cluster 1	Cluster 2	Cluster 3	Cluster 4	Cluster 5	TOT/Mn
**BHS**							
N	139	299	40	76	20	0	574
PI	1.16	3.67	9.04	12.58	11.29	–	4.88
INC	12.09	9.67	8.45	7.17	7.17	–	9.75
TDT	13.24	13.34	17.50	19.75	18.47	–	14.64
***S. aureus***							
N	522	827	132	87	42	0	1610
PI	1.40	3.78	9.26	13.35	15.84	–	4.29
INC	19.74	18.16	16.54	12.61	10.42	–	18.04
TDT	21.14	21.94	25.80	25.96	26.26	–	22.33
***E. coli***							
N	612	973	176	215	96	17	2089
PI	1.52	4.00	9.80	13.15	12.87	21.05	5.25
INC	14.06	12.70	9.19	10.09	11.18	13.45	12.47
TDT	15.57	16.70	19.00	23.24	24.06	34.51	17.72
***CoNS***							
N	486	652	88	56	28	5	1315
PI	1.64	3.87	9.20	11.31	15.92	17.66	4.03
INC	26.01	26.60	30.02	29.26	25.02	21.99	26.67
TDT	27.65	30.47	39.23	40.57	40.94	39.65	30.70
***Enterococcus* spp**							
N	322	232	48	30	7	8	647
PI	1.84	4.15	10.29	12.22	22.03	18.26	4.20
INC	17.85	15.41	12.25	13.25	8.04	6.01	16.09
TDT	19.68	19.56	22.53	25.47	30.07	24.28	20.29
***K. pneumoniae***							
N	183	224	45	53	11	0	516
PI	1.48	4.14	9.99	11.44	14.60	–	4.68
INC	16.94	14.73	14.07	11.06	13.31	–	15.05
TDT	18.43	18.87	24.06	22.50	27.91	–	19.73
**VGS/ANG**							
N	118	275	25	20	5	0	443
PI	1.38	3.80	8.91	15.72	10.01	–	4.19
INC	17.60	21.10	19.02	21.27	17.90	–	19.97
TDT	18.98	24.90	27.93	36.99	27.91	–	24.08
***S. pneumoniae***							
N	83	107	23	20	9	4	246
PI	1.91	3.67	8.32	10.75	12.66	6.49	4.46
INC	12.89	12.11	11.78	10.54	11.85	7.13	12.12
TDT	14.79	15.78	20.09	21.30	24.51	13.63	16.58
**Other coliforms**							
N	219	320	41	55	17	7	659
PI	2.39	4.09	10.13	13.16	12.96	17.63	5.03
INC	16.23	14.31	13.60	14.01	19.93	13.38	15.01
TDT	18.62	18.40	23.73	27.17	32.89	31.01	20.04
**NF**							
N	97	82	15	11	12	0	217
PI	1.71	3.99	8.42	14.33	12.48	–	4.27
INC	21.89	24.64	15.44	31.47	9.91	–	22.31
TDT	23.60	28.63	23.85	45.80	22.40	–	26.58

N: number of isolates, PI: preincubation time, BHS: beta-hemolytic *Streptococcus*, CoNS: coagulase-negative *Staphylococcus*, VGS/ANG: viridans group *Streptococcus* and *S. anginosus* group, Mn: mean, NF: non-fermenting gram negative bacilli, TOT: total.

**Table 3:** Statistical analysis of positivity rates for various bacterial species or groups stratified by preincubation time.

PI	N (%)	Chi-Sq	CATT	OR (95% CI)	*P* value	OR/h (95% CI)
**BHS**						
< 5 h	401 (5.41)	0.0807	0.0239	1		1.02 (1.00–1.03) *P* = 0.0650
5–10 h	106 (6.87)			**1.29 (1.03–1.61)**	**0.0244**	
10–20 h	54 (6.62)			1.24 (0.92–1.66)	0.1543	
> 20 h	13 (6.95)			1.30 (0.74–2.31)	0.3616	
***S. aureus***						
< 5 h	1243 (16.79)	**0.004**	**0.006**	1		**0.99 (0.98–1.00)** ***P* = 0.0468**
5–10 h	219 (14.20)			**0.82 (0.70–0.96)**	**0.0126**	
10–20 h	131 (16.05)			0.95 (0.78–1.15)	0.5937	
> 20 h	17 (9.09)			**0.50 (0.30–0.82)**	**0.0062**	
***E. coli***						
< 5 h	1467 (19.81)	** < 0.0001**	** < 0.0001**	1		**1.04 (1.03–1.04) ** ***P* < 0.0001**
5–10 h	321 (20.82)			1.06 (0.93–1.22)	0.3701	
10–20 h	236 (28.92)			**1.65 (1.40–1.94)**	** < 0.0001**	
> 20 h	65 (34.76)			**2.15 (1.59–2.93)**	** < 0.0001**	
***CoNS***						
< 5 h	1013 (13.68)	**0.0007**	**0.0007**	1		**0.97 (0.96–0.99) ** ***P* = 0.0001**
5–10 h	211 (13.68)			1.00 (0.85–1.17)	0.9986	
10–20 h	72 (8.82)			**0.61 (0.48–0.79)**	**0.0001**	
> 20 h	19 (10.16)			0.71 (0.44–1.15)	0.1672	
***Entero-coccus* spp**						
< 5 h	497 (6.71)	0.4658	0.113	1		0.99 (0.97–1.00) *P* = 0.0872
5–10 h	95 (6.16)			0.91 (0.73–1.15)	0.428	
10–20 h	45 (5.51)			0.81 (0.59–1.11)	0.1916	
> 20 h	10 (5.35)			0.79 (0.41–1.49	0.4616	
***K. pneumoniae***						
< 5 h	363 (4.90)	0.1493	0.0845	1		1.01 (0.99–1.02) *P* = 0.4418
5–10 h	94 (6.10)			1.26 (1.00–1.59)	0.0532	
10–20 h	50 (6.13)			1.27 (0.93–1.72)	0.1292	
> 20 h	9 (4.81)			0.98 (0.49–1.93)	0.9551	
**VGS/ANG**						
< 5 h	337 (4.55)	0.1056	0.3110	1		0.98 (0.96–1.01) *P* = 0.1461
5–10 h	83 (5.38)			1.19 (0.93–1.53)	0.161	
10–20 h	27 (3.31)			0.72 (0.48–1.07)	0.103	
> 20 h	6 (3.21)			0.70 (0.31–1.58)	0.3851	
***S. pneumoniae***						
< 5 h	179 (2.42)	0.1280	0.6289	1		1.00 (0.97–1.02) *P* = 0.8620
5–10 h	49 (3.18)			1.32 (0.96–1.83)	0.0859	
10–20 h	16 (1.96)			0.81 (0.48–1.35)	0.4166	
> 20 h	2 (1.07)			0.44 (0.11–1.77)	0.2462	
**Other coliforms**						
< 5 h	472 (6.37)	0.0922	**0.0190**	1		**1.02 (1.01–1.03) ** ***P* = 0.0058**
5–10 h	102 (6.61)			1.04 (0.83–1.30)	0.7266	
10–20 h	68 (8.33)			**1.34 (1.02–1.74)**	**0.0327**	
> 20 h	17 (9.09)			1.47 (0.88–2.44)	0.1375	
**NF**						
< 5 h	166 (2.24)	0.1450	0.5999	1		0.99 (0.96–1.02) *P* = 0.4570
5–10 h	27 (1.75)			0.77 (0.52–1.17)	0.2285	
10–20 h	23 (2.82)			1.27 (0.81–1.97)	0.298	
> 20 h	1 (0.53)			0.23 (0.03–1.68)	0.1492	

*P* values for Chi-square and the Cochran-Armitage trend test are shown. Logistical regression analysis was performed both with stratified preincubation times, using the < 5 h preincubation group as reference, and using time as a continuous variable. Statistically significant results are bolded. PI: preincubation, CI: confidence interval, Chi-sq: Chi-square, CATT: Cochran-Armitage Trend Test, OR: odds ratio. N: number of isolates, BHS: beta-hemolytic *Streptococcus*, CoNS: coagulase-negative *Staphylococcus*, VGS/ANG: viridans group *Streptococcus* and *S. anginosus* group, Mn: mean, NF: non-fermenting gram negative bacilli, TOT: total.

### Prospective recovery of undetected bacterial growth

A total of 451 bottles were prospectively assessed by terminal subculture and molecular assays (Table S3), including 207 blood bottles with preincubation times < 12 h, used as controls to establish baseline contamination rates. This was done in order to assess both for undetected growth due to loading of blood culture bottles in the automated blood culture system after bacteria had exited their exponential phase of growth, and for the presence of genetic material derived from non-viable bacteria which may not have replicated after the preincubation period. The control group showed the highest rate of growth on subcultures with 4.83% (10 bottles). Half of these bottles had growth of coagulase-negative *Staphylococcus* species, as listed in Table S3. Other species isolated included *Cutibacterium acnes* (2 bottles), *Bacillus* sp (1 bottle; non-*anthracis*, non-*cereus*), *Streptococcus oralis* (1 bottle) and *Candida albicans* (1 bottle). Overall, 16 blood culture bottles were positive for growth on subculture, and in all cases only between 1 to 3 colonies were seen on the plates, in between streaking lines or adjacent to the side of the plate, and outside of the first streaking quadrant, with three exceptions:

- one bottle with moderate growth of *C. albicans* (control group)
- one culture plate growing *B. cereus* (15–18 h group) with 20 colonies seen in the path of a drop of condensation with no colonies seen on the inoculation streaks
- one culture plate with mixed growth (21 h group), located off the inoculation streaks in the 3rd and 4th quadrant of the chocolate agar plate

Amplification of genetic material was noted in one blood culture bottle using an assay targeting *Staphylococcus* spp. Decision to use this assay was made when we noted that there were no isolates of *S. aureus* recovered from the cluster 5 site (Table 2). A positive reaction was noted with the *Staphylococcus* 16S probe, but not with the *nuc* and *mecA/mecC* probes, indicating the presence of a methicillin-susceptible coagulase-negative *Staphylococcus* isolate. Universal 16S RNA amplification from this bottle also showed detectable signal.

## Discussion

With regards to laboratory services consolidation, expert opinion recommends localized provision of tests with expected turn-around-times of less than 4 h as well as tests for which delays in generating results would negatively impact patient care^1^. This includes blood cultures, with current recommendations for incubation within 2 to 4 h of collection. Not only does this limit opportunity for transportation, but even sites that have in-house microbiology struggle to fulfill this parameter. In a previous study showing that an increase in transport time is partially mitigated by a decrease in incubation time^3^, an interquartile (IQR) range of 2 to 15 h of transport was noted for cultures originating from the site where the main laboratory was located. An earlier study in a tertiary care hospital also showed mean transportation time from ward to laboratory of 10.4 h^14^, and a subsequent study at the same site yielded a median transport time of 3.52 h, with an IQR of 1.33 to 13.90 h^15^. Our own findings, based on the audit of a very large dataset, generally reflect those observed in previous studies: preincubation times varied greatly between sites, a general trend towards decreasing time to detection was noted with an increase in preincubation time, but this did not fully compensate for the lengthy preincubation and led to an overall increase in total time to detection. Only the testing site and cluster 1 sites fulfilled, on average, the 4 h guideline for preincubation: inoculated blood culture bottles reached the main laboratory promptly, with an IQR of 0.64 to 1.72 h, which compares favorably to previous studies^14,15^. Overall, the preincubation time data we have gathered here has prompted our organization to reassess courier routes, with an attempt being made to optimize the use of regional hubs and transport of cultures on an “as needed” basis for distant sites for which one daily pick up in the setting of low, unpredictable volumes, may not be optimal.

While distance may very well be the main factor explaining the differences seen in preincubation times, other variables are at play including the number of daily couriers and operating hours of the peripheral sites’ laboratories, which are involved in the reception of the samples and their preparation for shipping. The data from sites which seemed to have worse preincubations times than what would be expected based on distance alone (specifically those sites falling above the trendline on Fig. 1) was reviewed, and the total volume of blood culture bottles collected for each hour of the day was plotted along with the mean preincubation time for blood culture bottles collected at specific times for each site (data not shown). In most instances courier pick up times were not synchronized with periods of higher volumes of blood culture collections. In some instances, these periods of higher volumes occurred after the local laboratory had closed, worsening delays. Modification of opening hours or strategies for handling of blood culture bottles after hours will need to be devised to allow for optimal management of these clinically sensitive samples.

The main concerns surrounding prolonged preincubation times relate to the fragility of some bacterial species which may affect recovery or lead to potential overgrowth during preincubation, and thus may impact growth detection post-incubation. There has been a previous report of false negative results for *Pseudomonas aeruginosa, Candida albicans, Acinetobacter baumanii* and *Stenotrophomonas maltophilia* possibly due to overgrowth during preincubation prior to incubation in a BacT/ALERT system^16^, findings which were not reproduced subsequently in a continuous-monitoring system with upgraded culture media^17^. Another study showed a statistically significant increase in false negative results after 24 h of preincubation either at room temperature or 35 ºC for *A. baumanii*, *B. fragilis*, *E. coli*, *E. faecalis, P. aeruginosa* and *S. pneumoniae*, and this seemed to occur more frequently with the BACTEC 9240 continuous-monitoring system than with the BacT/ALERT 3D system^18^. This study also showed no significant increase in false negative results for the first 12 h of preincubation at room temperature. Lee & al. also showed increased false negative results for *P. aeruginosa* but not *S. aureus* and *E. coli* in a simulated bacteremia experiment following preincubation at 25 ºC or 37 ºC for 48 h and 24 h respectively, again with the BacT/ALERT system^19^. Finally, detection of fastidious organism following delayed-entry into the BacT/ALERT continuous-monitoring system was also assessed, with a small difference in detection rates noted between direct insertion (92.5%) and preincubation for 24 h at room temperature (90%) at low concentration of inoculum^20^. Only the recovery of *Streptococcus sanguinis* was affected following preincubation in this condition, with good recovery of *S. pneumoniae*.

Given the above, the lack of recovery of *S. aureus* and beta-hemolytic *Streptococcus* spp, which are common agents of community-acquired skin and soft tissue or joint infections with potential for secondary bacteremia, from the site in cluster 5 was a major cause for concerns. While it is reassuring that four blood cultures with *S. pneumoniae*, which is considered more fastidious than other bacteria due to its tendency to autolyze, were recovered from that site, these bottles were collected at the same time on the same patient and had preincubation times of 6.49 h only, well below the mean preincubation time for this distant site. Statistical analysis seemed to confirm our concerns, with significant lower odds ratio for recovery of *Staphylococcus* species, including *S*. *aureus*, with each additional hour of preincubation. A statistically significant lower OR for recovery of *S. aureus* was also noted with 5–10 h of preincubation, but clinical relevance of this signal is unclear given that this was not noted for the 10–20 h preincubation group. It should be noted that a lower number of *S. aureus* isolates, but not a complete absence, may be expected from smaller community centers: patients are typically less medically complex, with lower absolute number of intravascular device-associated infections which are commonly seen in tertiary centers. This would lead to an increase in the proportion of cases of bacteremia caused by *S. aureus* in larger care centers and as such it is possible that this might have introduced a bias in our logistic regression analysis: community centers would be expected to have a lower proportion of cases of bacteremia linked to *S. aureus* or CoNS, and since they are located farther way from the testing site these would preferentially be included in higher preincubation time clusters. This bias might also explain why logistic regression also showed greater odds of recovery of *E. coli* and other *Enterobacterales* with increasing preincubation times as bacteremia with these organisms may represent a greater share of positive blood culture from peripheral, community sites in the context of a higher proportion of cases of secondary bacteremia due to urinary tract infections.

Terminal subcultures mainly yielded growth of organisms generally recognized as contaminants except one case of moderate growth of *Candida albicans* from an aerobic blood culture bottle from the control group. This was surprising given that there is no known limitation for the detection of *C. albicans* in BACTEC FX even at low inoculums, with blood cultures flagged as positive within 24.5 h of insertion into the instrument on average^21^. Careful clinical and microbiological review done at the time showed a preincubation time of 88 min, did not show evidence of antifungal treatment prior to the collection of the blood culture, and no evidence of symptoms concerning for ongoing, untreated fungemia. The initial blood culture result was nonetheless corrected and, the primary care providers were made aware of this unexpected finding to allow for appropriate clinical correlation. Antifungal treatment was not initiated thereafter indicating that this result was felt to represent contamination. It should be noted that the molecular assays we used to assess for undetected growth of non-viable organisms was targeting bacterial agents only and would not have been adequate to assess for the presence of fungal agents. Our *Staphylococcus* spp molecular assay led to the identification of one blood culture bottle which may have contained a methicillin-susceptible coagulase-negative *Staphylococcus* isolate, and which was not identified by terminal subculture. This same bottle showed detectable signal by 16S rRNA amplification, and this was felt to represent the same CoNS isolate. Of note, this blood culture bottle was from the control group, with a preincubation time < 12 h. None of our molecular assays identified previously undetected growth of pathogenic bacterial isolates, which also provided reassurance that preincubation did not prevent bacterial recovery in our study.

Other limitations than the ones already stated may have impacted our study. First, while standard practice in our tertiary care institution is to collect blood culture bottles prior to initiation of antibiotherapy, we cannot confirm if this practice was enforced in our smaller community hospitals. This could have introduced a bias in the recovery of organisms for sites located further from the testing site and contributed to the lack of recovery of *Staphylococcus* spp and beta-hemolytic *Streptococcus* from the cluster 5 site. Second, while our study included pediatric blood culture bottles, the vast majority would have come cluster 1 site E, a regional tertiary care pediatric center located within walking distance of the testing site. Subgroup analysis to assess bacterial recovery as a function of time for pediatric blood culture bottles was therefore not possible, but this subgroup would not be expected to affect our main conclusions. Thirdly, the prevalence of specific pathogens in each cluster sites prior to regionalization could not be assessed as this data, largely based on paper records and laboratory worksheets, was not available to the research team. As prevalence of specific pathogens could vary over time, as an example in the context of outbreaks, comparison with historical data may not have allowed to infer success or lack-thereof microbial recovery post-regionalization. Lastly, we only recovered 101 anaerobic isolates and 89 *Neisseria* spp or *Haemophilus* spp isolates, small numbers which precluded analysis across all sites.

Overall, the results from our study indicate that an increase in preincubation time does impact total detection time, and that bacterial recovery may be impacted for some species with a prolonged preincubation although the presence of a possible bias in the logistic regression analysis should be considered, and results from terminal subcultures and molecular assays did not support this finding. Based on the prolongation in total detection time alone and the associated clinical impact of a delayed report, centralized laboratories should aim at reducing preincubation times as much as possible through optimization of courier services and processing of collected samples at peripheral sites. However, given the new reality of a centralization of microbiological services in the context of budget constraints, we feel that our data does support considering a modest increase in the currently recommended maximum preincubation times to 8 to 12 h.

## Methods

### Study sites and samples

EORLA RMRL is located within the General campus of The Ottawa Hospital, a tertiary care academic center, and provides diagnostic microbiology services 24 h a day, 7 days a week, in eastern Ontario. Seven hospitals are located 0.5 to 12.3 km from RMRL within the city of Ottawa, including a pediatric tertiary care centre, a specialized centre for cardiac interventions, a large tertiary care centre with a trauma centre, a large tertiary care centre with an hematology and stem cell transplant ward, an outpatient hospital housing a renal transplant outpatient clinics and one-day elective surgery, and two community hospitals offering secondary care services. Twelve smaller community hospitals are located within 49.6 to 197 km of the RMRL. In order to facilitate interpretation of the results, sites were grouped into clusters (Table 1) based on preliminary data showing similarities in mean and median preincubation times and compared to the testing site; these sites often share common courier routes for sample delivery. Cluster 1 is composed of hospitals located within the city of Ottawa, other than the testing site. Cluster 2 includes two large community hospitals located east of Ottawa. Cluster 3 includes three community hospitals located west of Ottawa and one community hospital located to the south. Cluster 4 is composed of four community hospitals located in suburban areas west and south of Ottawa, and one smaller site located farther west. Cluster 5 is composed of a single site serving a distant, small community, for which mean and median preincubation times were outliers when compared to other sites.

Between July 1st 2017 and June 30th 2018, a total of 70,458 inoculated blood culture bottles were received at the RMRL (Table 1). Included were aerobic, anaerobic and pediatric blood culture bottles (BACTEC™ Plus Aerobic/F, BACTEC™ Lytic/10 Anaerobic/F and BACTEC™ Peds Plus/F culture vials respectively, BD-Canada, Mississauga, ON). For the purpose of this study, the following exclusion criteria were established:

- bottles for which no loading time, defined as the time point at which the bottle was inserted into the BACTEC instrument, was recorded
- bottles for which time from collection to loading was inferior to the minimum expected transport time from the collecting ward to the testing site, established using Google Maps, under normal traffic conditions. This was established in order to exclude bottles for which the collection time, which is a mandatory information in our laboratory information system, was not recorded at the collection site and instead recorded as the reception time at the testing site.
- bottles originating from autopsies
- bottles originating from proficiency testing panels

This left a total of 69,604 blood cultures bottles (59,077 negatives, 10,527 positives), all of which were incubated in the BACTEC FX continuous-monitoring system for 5 days as per our standard blood culture protocol. Pediatric blood bottles were included in the analysis. Pediatric blood bottles were included in the analysis, and mainly came from Cluster 1 Site E (pediatric hospital, 540 negative and 60 positive bottles). Positivity rates and contamination rates, defined as the recovery of skin organisms (coagulase-negative *Staphylococcus*) or other common environmental organisms (*Paenibacillus* spp*, Bacillus* spp non-*anthracis*) in single bottles from a set of blood cultures, were also documented for each site.

### Preincubation time, incubation time (INC) and total detection time (TDT)

Preincubation time, i.e. the time from drawing of the blood sample on the patient to the insertion of the inoculated bottle into the continuous monitoring system, was calculated for each bottle. We included data from negative bottles in the calculation of median and mean preincubation times for all individual sites. Only monomicrobial positive blood cultures were used to establish INC (defined as time from insertion into the automated blood system to growth detection) and TDT (defined as time from collection of samples to growth detection) in order to allow analysis by species clusters. INC was calculated as the time and date at which the bottle was flagged positive (detection time) minus the time and date of insertion into the BACTEC FX. TDT was calculated as the detection time minus the time and date of drawing. Whenever the detection time was not available, this was inferred from the time at which the gram stain result was recorded in the laboratory information system minus 30 min, in order to account for the time required to prepare, stain and read the slides.

### Data visualization and statistical analysis

Preincubation time and TDT data was presented visually using the “box and whisker” chart type in Microsoft Excel (Figs. 2, 3). Data points falling more than 1.5 times the interquartile range above the third quartile or below the first quartile were considered outliers. For the statistical analysis of positivity rates by bacterial types and site clusters, all *P* values are reported as two-sided. Hypothesis testing was carried out with an overall level of significance set using a *P* value < 0.05. Median and average preincubation times were established for all sites, including the testing site. The one-way analysis of variance (ANOVA) test was used to compare preincubation times, INC and TDT between cluster sites. Pearson correlation coefficient was applied to assess for a possible correlation between preincubation time and INC for positive samples. This was further investigated with one-way ANOVA test by clustering samples based on preincubation time (< 5 h, 5 to 10 h, 10 to 20 h, > 20 h). Positivity rates for each time clusters, both for overall positivity and positivity by bacterial subsets (beta-hemolytic *Streptococcus* (BHS), *S. aureus*, *Escherichia coli*, Coagulase-negative *Staphylococcus* (CoNS), *Enterococcus* spp, *Klebsiella pneumoniae*, *Viridans* group *Streptococcus / S. anginosus* group (VGS/ANG), *S. pneumoniae*, *Enterobacterales* other than *E. coli* and *K. pneumoniae*, and non-fermenting gram negative bacilli (NF)), were further compared using the Chi-square test and the Cochran-Armitage Trend Test to assess for a trend across the ordinal categories of collection time. Logistic regression models were also used and applied to the previously defined time clusters, but also using preincubation time as a continuous variable in order to define the relationship between preincubation time and growth outcomes with odds ratio with 95% confidence intervals. All data manipulations and statistical analyses were performed using Statistical Analysis System, Version 9.3 (SAS Institute Inc., Cary, North Carolina, USA).

### Prospective assessment of undetected growth in blood culture bottles

From July 2019 to October 2019, we conducted a prospective analysis of inoculated blood culture bottles for which a preincubation time > 12 h and a lack of growth detection following standard incubation in the BACTEC FX were recorded. This time point was chosen as previous study had shown no loss of viability for various micro-organisms held at room temperature for that amount of time^5,18^. Preincubation intervals of 12 to 15 h, 15 to 18 h, 18 to 21 h and > 21 h were established, with 56–98 bottles selected for each time clusters depending on availability (Supplementary data, Table S3). A total of 207 bottles with incubation times < 12 h were selected as controls. Overall, 500 blood culture bottles were tested. All remote sites were included in the sampling. Bottles with preincubation of > 12 h were flagged upon reception at the testing site, and subsequently incubated in the BACTEC FX as per the manufacturer’s instruction. Flagged bottles that exhibited no growth after 5 days of incubation were retrieved. These were subcultured to chocolate agar plates for incubation under aerobic conditions (48 h incubation at 35ºC under 5% CO_2_ atmosphere) and to CDC agar plates for incubation under anaerobic conditions (48 h incubation at 35ºC) as per our standard blood culture protocol. Aliquots of blood were simultaneously retrieved for molecular testing (detection of *Staphylococcus* spp and 16S RNA amplification described below). No gram stain was performed. Aerobic plates were incubated in Kiestra ReadA Compact smart incubators (Becton, Dickinson & Company, Franklin Lake, NJ), and plates were imaged at 24 and 48 h. Anaerobic plates were incubated in anaerobic jars in standard incubators, but also imaged on the ReadA Compact after 48 h. The presence or absence of growth was recorded. Since these were clinical samples, any plate exhibiting growth was rerouted to the technologist performing work up for positive blood cultures, and bacterial identification and susceptibility testing was performed as per our standard clinical protocol, when appropriate. Identification was performed by MALDI-TOF MS (Bruker Ltd, Milton, ON) using Biotyper v.7854.

### Molecular detection of *Staphylococcus* spp and universal 16S RNA amplification

Aliquots of 1 ml of blood culture broth were retrieved from bottles with prolonged preincubation (> 12 h) and absence of growth in BACTEC FX at 5 days and tested using a laboratory developed assay for the qualitative detection of *Staphylococcus* spp*, S. aureus* and MRSA through the simultaneous amplification of genes encoding the *Staphylococcus* 16S rRNA, the *S. aureus* endonuclease *nuc* and the MRSA *mecA/mecC*. This sample was also tested for the presence of bacterial 16S RNA as previously described^22^. Six microliters of blood culture were added to a MagNA Lyser Green Bead vial (Roche Diagnostics) containing 600 µL of TE buffer. The mix was then lysed in a MagNA Lyzer instrument for 70 s at 5,000 rpm. 2 μL of blood extract was used directly for real-time PCR. Specific primers and probes sequences for *mecA/mecC* and *nuc* were previously described^23^, as were the primers and probe sequences for *Staphylococcus* spp 16S rRNA^24^ and the universal 16S RNA primers^22^ (Supplementary data, Table S1). For the latter assay, which was developed for molecular detection of micro-organisms directly from clinical samples, published data indicate a limit of detection of 150 CFU per PCR reaction from blood sample^22^, while internal validation showed a limit of detection of 75.5–85 CFU in sterile fluids and tissue samples respectively (data not shown).

Real-time PCR was conducted in a total volume of 10 μL, containing 1 × QuantiFast Multiplex PCR Kit (QIAGEN), MecA, MecAlga251, Nuc, Staph16S primers and corresponding dual-labelled probes (Bioresearch Technologies) as per the final concentrations listed in Table S1. Real-time PCR was carried out using a LightCycler 2.0 system (Roche Diagnostics). Amplification consisted of Taq polymerase activation at 95ºC for 5 min followed by 40 cycles of 20 s denaturation at 95ºC and 60 s annealing at 58ºC. Positive controls with methicillin-resistant *S. aureus* (MRSA ATCC 4433000), methicillin-susceptible *S. aureus* (MSSA ATCC 25923), methicillin-susceptible coagulase-negative *Staphylococcus* (ATCC 12228) and a negative control with sterile distilled water (DW) were included throughout the procedures. Probes for *mecA/meC*, *nuc* and 16S rRNA were detected using channels 530, 560 and 610 respectively. A combination of signal detection in all three channels is interpreted as indicating the presence of MRSA in the clinical sample tested, while a positive signal in channels 560 and 610 (*nuc* and *Staphylococcus* spp 16S rRNA) is interpreted as indicating the presence of *S. aureus* in the clinical sample. Isolated signal in channel 610 is indicative of coagulase-negative *Staphylococcus* spp*.* The same blood extract used for the *Staphylococcus* spp multiplex qPCR was used for monoplex qPCR with universal 16S primers 27F and 1RR-B and 514S dual-labelled probes (Table 1), using the same PCR conditions as above.

### Ethics statement

This study did not involve the collection of clinical information or modification to standard of care practices. As a quality improvement project, it did not require review by the OHRI Research Ethics Board.

## Supplementary Information

Supplementary Information.

## Data Availability

The datasets generated and/or analysed during the current study are available from the corresponding author on reasonable request.

## Abbreviations

ANOVA: Analysis of variance
CHEO: Children’s Hospital of Eastern Ontario
DNA: Deoxyribonucleic acid
EORLA: Eastern Ontario Regional Laboratory Association
INC: Incubation time
MALDI-TOF MS: Matrix-assisted laser desorption/ionization time-of-flight mass spectrometry
MRSA: Methicillin-resistance *Staphylococcus aureus*
PCR: Polymerase chain reaction
RMRL: Regional Microbiology Reference Laboratory
RNA: Ribonucleic acid
TDT: Total detection time

## References

[1] Sautter RL, Thomson RB (2015). Consolidated clinical microbiology laboratories. J. Clin. Microbiol..

[2] Vandenberg O, Kozlakidis Z, Schrenzel J, Struelens MJ, Breuer J (2018). Control of infectious diseases in the era of european clinical microbiology laboratory consolidation: New challenges and opportunities for the patient and for public health surveillance. Front. Med..

[3] Rönnberg C, Mildh M, Ullberg M, Özenci V (2013). Transport time for blood culture bottles: Underlying factors and its consequences. Diagn. Microbiol. Infect. Dis..

[4] Saito T (2009). Delayed insertion of blood culture bottles into automated continuously monitoring blood culture systems increases the time from blood sample collection to the detection of microorganisms in bacteremic patients. J. Infect. Chemother..

[5] Sautter RL (2006). Effects of delayed-entry conditions on the recovery and detection of microorganisms from BacT/ALERT and BACTEC blood culture bottles. J. Clin. Microbiol..

[6] Janapatla RP (2010). Effect of overnight storage of blood culture bottles on bacterial detection time in the BACTEC 9240 blood culture system. J. Microbiol. Immunol. Infect..

[7] Berild D, Mohseni A, Diep LM, Jensenius M, Ringertz SH (2006). Adjustment of antibiotic treatment according to the results of blood cultures leads to decreased antibiotic use and costs. J. Antimicrob. Chemother..

[8] Byl B (1999). Impact of infectious diseases specialists and microbiological data on the appropriateness of antimicrobial therapy for bacteremia. Clin. Infect. Dis..

[9] Barenfanger J (2008). Decreased mortality associated with prompt gram staining of blood cultures. Am. J. Clin. Pathol..

[10] Gilligan, P. H., Alby, K. & York, M. K. Blood Cultures: General Detection and Interpretation. In *Clinical Microbiology Handbook* 4th edn, vol. 1 (ed. Leber AL) Washington, DC: (ASM Press, 2016).

[11] Baron, E. J. Specimen Collection, Transport, and Processing: Bacteriology. In *Manual of Clinical Microbiology* 11th edn, vol. 1 (eds. Jorgensen JH et al.,) Washington, DC: (ASM Press, 2015).

[12] CLSI. Principles and Procedures for Blood Cultures; Approved Guideline. *CLSI document M47-A**.* (Clinical and Laboratory Standards Institute, 2007).

[13] Kirn TJ, Weinstein MP (2013). Update on blood cultures: How to obtain, process, report, and interpret. Clin. Microbiol. Infect..

[14] Kerremans JJ (2008). Rapid identification and antimicrobial susceptibility testing reduce antibiotic use and accelerate pathogen-directed antibiotic use. J. Antimicrob. Chemother..

[15] Kerremans JJ, van der Bij AK, Goessens W, Verbrugh HA, Vos MC (2009). Needle-to-Incubator transport time: Logistic factors influencing transport time for blood culture specimens. J. Clin. Microbiol..

[16] Klaerner H-G (2000). Failure of an automated blood culture system to detect nonfermentative gram-negative bacteria. J. Clin. Microbiol..

[17] Seegmüller I, Eschenbach U, Kamereck K, Miethke T (2004). Sensitivity of the BacT/ALERT FA-medium for detection of Pseudomonas aeruginosa in pre-incubated blood cultures and its temperature-dependence. J. Med. Microbiol..

[18] Akan ÖA, Yıldız E (2006). Comparison of the effect of delayed entry into 2 different blood culture systems (BACTEC 9240 and BacT/ALERT 3D) on culture positivity. Diagn. Microbiol. Infect. Dis..

[19] Lee D-H (2013). Growth dynamics of *Staphylococcus aureus*, *Escherichia coli*, and *Pseudomonas aeruginosa* as a function of time to detection in BacT/Alert 3D blood culture bottles with various preincubation conditions. Ann. Lab. Med..

[20] Wilms MC, Stanzel S, Reinert RR, Burckhardt I (2009). Effects of preincubation temperature on the detection of fastidious organisms in delayed-entry samples in the BacT/ALERT 3D blood culture system. J. Microbiol. Methods.

[21] Menchinelli G (2019). In vitro Evaluation of BACT/ALERT® VIRTUO®, BACT/ALERT 3D®, and BACTEC^TM^ FX Automated Blood Culture Systems for Detection of Microbial Pathogens Using Simulated Human Blood Samples. Front. Microbiol..

[22] Schabereiter-Gurtner C (2008). Evaluation of a protocol for molecular broad-range diagnosis of culture-negative bacterial infections in clinical routine diagnosis. J. Appl. Microbiol..

[23] Pichon B (2012). Development of a real-time quadruplex PCR assay for simultaneous detection of nuc, Panton-Valentine leucocidin (PVL), mecA and homologue mecALGA251. J. Antimicrob. Chemother..

[24] Kim J-U, Cha C-H, An H-K, Lee H-J, Kim M-N (2013). Multiplex real-time PCR assay for detection of methicillin-resistant *Staphylococcus aureus* (MRSA) strains suitable in regions of high MRSA endemicity. J. Clin. Microbiol..

