# Supplementary material for: Effect of delayed entry of blood culture bottles in BACTEC automated blood culture system in the context of laboratory consolidation
Source: Sci Rep. 2022 Jan 25;12:1337. doi: 10.1038/s41598-022-05246-3 (PMC8789921; doi:10.1038/s41598-022-05246-3)
Supplement: Supplementary file 1 — Supplementary Information. [file 41598_2022_5246_MOESM1_ESM.pdf]

Effect of delayed entry of blood culture bottles in BACTEC automated blood culture system in the context of laboratory consolidation

V. Deslandes, Darya Rafipour, Ivan Gorn, Elham Sabri, N. Sant and M. Desjardins

SUPPLEMENTARY DATA

Table S1. PCR primers and TaqMan probes for mecA, NUC, staphylococcal 16S rRNA, and PCR primers for universal 16S rRNA.

Target genes	Sequence (5'-3')	Final Con (uM)
**mecA-F**	GGCAATATTAMCGCACCTCA	0.500
**mecA-R**	GTCTGCCASTTTCTCCTTGT	0.500
**mecA probe**	FAM-AGATCTTATGCAAACTTAATTGGCAAATCC-BHQ1	0.125
**mecAlga251-F**	AGATTTAAAGTAGTAGACGGCA	0.500
**mecAlga251-R**	TTTCACCGATTCCCAAATCT	0.500
**mecAlga251 probe**	FAM-TTTGCCCGCATTGCATTAGCATTAGGA-BHQ-1	0.125
**Nuc-F**	CATCCTAAAAAAGGTGTAGAGA	0.500
**Nuc-R**	TTCAATTTTMTTTGCATTTTCTACCA	0.500
**Nuc probe**	HEX-TTTTCGTAAATGCACTTGCTTCAGGACCA-BHQ-1	0.250
**Staph16SrRNA-F**	CTTACCAAATCTTGACATCCTTT	0.500
**Staph16SrRNA-R**	CTCGTTGCGGGACTTAAC	0.500
**Staph16SrRNA probe**	CAL610-CGTCAGCTCGTGTCGTGAGAT-BHQ-2	0.250
**Universal 27F**	AGA GTT TGA TCM TGG CTC AG	0.500
**Universal 16S1RR-B**	CTTTACGCCCARTRAWTCCG	0.500
**Univesal 514S probe**	FAM-TNTTACCGCGGCTGCTGGCACG-BHQ-1	0.250

Table S2. Mean time to detection (TTD) (hours) and positivity rate following stratification of blood cultures as a function of preincubation time. Results for statistical analysis of positivity rates using Chi-square, the Cochran-Armitage trend test and logistical regression analysis are shown. PI: preincubation, N NEG: number of negative samples. N POS: number of positive samples, Mn: mean, CI: confidence interval, SD: standard deviation, Md: median, LQ: lower quartile, UQ: upper quartile, Min: minimum, Max: maximum, Chi-sq: Chi-square, CATT: Cochran-Armitage Trend Test, OR: odds ratio.

PI	N NEG	N POS (%)	TTD (95% CI)	SD	Md	LQ	UQ	Min	Max	Chi-Sq P-value	CATT P-value	Logistical Regression	
												OR (95% CI)	P-value
**< 5 hours**	51393	7846 (15.27%)	20.58 (20.18 – 20.97)	17.89	14.15	10.7	22.28	1.41	132.63	0.307	0.089	1	-
**5 – 10 hours**	11004	1620 (14.72%)	19.22 (18.32 – 20.12)	18.48	12.32	9.27	21.28	2.26	129.45			0.958 (0.904 - 1.015)	0.148
**10 – 20 hours**	5818	865 (14.87%)	16.07 (14.87 – 17.27)	17.99	9.72	6.17	17.5	1.51	121.80			0.969 (0.898 – 1.046)	0.422
**> 20 hours**	1389	196 (14.11%)	16.14 (13.32 – 18.96)	20.02	8.25	4.29	19.65	1.19	105.76			0.912 (0.783 – 1.063)	0.237

Table S3. Prospective recovery of undetected bacterial growth. Blood culture bottles were groups by preincubation time clusters (<12 hours, 12-15 hours, 15-18 hours, 18-21 hours, and > 21hours), with the <12 hours group being used as control. Total number of positive subcultures, with rates and results from molecular studies are shown, along with bacterial identifications. TOT: total, SUB: number of positive bottles, MS: methicillin-susceptible, CoNS: coagulase-negative *Staphylococcus sp*

*: same bottle identified by *Staphylococcus sp* PCR

Group	TOT	POS	%	*Staph. PCR*	16s	Isolates:
**Ctrl**	207	10	4.83	1<br>(MS-CoNS)	1*<br>(*S. epidermidis)*	5x CoNS (2x *S. warneri,* 2x *S. epidermidis*, *S. capitis*)<br>2x *Cutibacterium acnes*<br>1x *Bacillus sp*<br>1x *Streptococcus oralis*<br>**1x *Candida albicans***
**12-15h**	74	1	1.35	0	0	1x *Bacillus sp*
**15-18h**	98	3	3.06	0	0	2x *Bacillus cereus,* 1x *S. epidermidis*
**18-21h**	65	2	3.08	0	0	1x *B. cereus*, 1x *Bacillus sp.*
**>21h**	56	1	1.79	0	0	1x *Bacillus sp.*
**TOT**	500	17	3.60	1	1	
